# Crossover clinical trial of pain relief in cataract surgery

**DOI:** 10.1007/s10792-017-0554-y

**Published:** 2017-06-20

**Authors:** Suvin Choi, Sang-Gue Park, Lorne Bellan, Hyung-Hwan Lee, Sung Kun Chung

**Affiliations:** 1 Da Vinci College of General Education, Chung-Ang University, Seoul, Republic of Korea; 2 Department of Applied Statistics, Chung-Ang University, Seoul, Republic of Korea; 3 Department of Ophthalmology, Misericordia Health Centre, Winnipeg, Canada; 4 School of Korean Music, Chung-Ang University, Anseong, Republic of Korea; 5 Saevit Eye Hospital, Goyang, Republic of Korea

**Keywords:** Cataract surgery, Pain, Crossover design, Washout period, Adjuvant therapy

## Abstract

**Purpose:**

To determine the effects of intra-operative Korean traditional music on pain experienced by Korean patients undergoing sequential bilateral cataract surgery.

**Methods:**

This was a two-sequence, two-period, and two-treatment crossover study. Fifty-two patients with cataracts were divided into two groups by block randomization, and bilateral cataract surgery was performed. In group 1, patients listened to Korean traditional music (KTM) during their first but not second cataract surgery. This sequence was reversed for patients in group 2. After each surgery, patients scored their pain intensity (PI) using a visual analog scale (VAS) ranging from 0 to 10, where 0 was ‘no pain’ and 10 was ‘unbearable pain.’

**Result:**

There was a statistically significant reduction in the mean VAS score with KTM (3.1 ± 2.0) compared to that without KTM (4.1 ± 2.2; *p* = 0.013). However, there were no statistically significant differences in blood pressure or pulse rates.

**Conclusion:**

KTM had a significant effect on reducing pain experienced by patients during cataract surgery. This may be useful in the context of other surgical procedures to reduce pain in Korean patients.

## Introduction

Loss of vision is one of the most feared disabilities [1], and cataracts are a major cause of vision loss, particularly in the elderly. Because of the rapid aging of the global population, the incidence of cataracts has increased substantially in recent years [2], and cataract surgery is now one of the most commonly performed procedures worldwide. The elderly are likely to have comorbidities, such as diabetes mellitus, hypertension, cardiac disease, and pulmonary disease [3]. Moreover, elderly patients with cataracts and comorbidities may be uniquely vulnerable and particularly sensitive to the stresses of trauma, hospitalization, surgery, and sedatives [4, 5] and are not able to cope with pain or anxiety [6].

Progressive improvements in cataract surgical techniques have resulted in a decline in intra-operative discomfort, allowing the stepwise change from general anesthesia to regional anesthesia (a block) to simple topical anesthesia. Although there are differing views regarding the use of anesthesia, surgeons have increasingly favored topical anesthesia as the default method for phacoemulsification [3, 6, 7].

Interestingly, music accompanied by verbal assurance as adjuvant treatment can be used in elderly patients, who are vulnerable to the side effects of sedatives [8]. Many studies have examined how music affects people’s thought processes and physical functions [9–11]. Guidelines in the USA recommend the use of music as an adjuvant to sedative medication for management of acute and postoperative pain [12]. Indeed, various reports have shown that introduction of relaxing music at the preoperative stage significantly reduces anxiety in patients undergoing minor surgical procedures compared with the administration of midazolam [8]. However, the efficacy of employing music for the purposes of pain relief and postoperative satisfaction in lieu of sedatives (e.g., midazolam and lorazepam) is controversial, and studies have shown conflicting results [9, 13, 14].

Studies on the effects of music in patients undergoing ophthalmic surgery have also shown varied outcomes. For example, Bellan et al. [5] reported that patients who had been administered oral sedatives and who listened to music showed decreased levels of anxiety and increased levels of sedation. Additionally, music was found to have a positive effect on lowering systolic blood pressure before and during cataract surgery [15]. Chen et al. [14] observed that music engagement decreases anxiety during intravitreal injection. Decreased systolic blood pressure and increased satisfaction were recorded among elderly patients undergoing cataract surgery with music intervention [16]. In general, both music and verbal reassurances are used as means of relieving patients’ anxiety and pain under anesthesia during surgery [9, 12]. However, to the best of our knowledge, most studies have examined the use of western-type classical music on pain and sedation. No reports have described the effects of Korean traditional music (KTM) on perceived pain in patients undergoing surgery.

Therefore, in this study, we examined the effects of KTM, playing throughout the entire procedure, on patient pain scores and blood pressure measurements during surgical procedures in delayed bilateral sequential cataract recipients.

## Materials and methods

### Sample size calculation

Sample size for this study was estimated with a 95% confidence interval, statistical power 80%, and effective size of 0.6, and the required sample size was 22 subjects using the following formula.

$${{n = \left( {z_{\alpha /2} + z_{\beta } } \right)^{2} } \mathord{\left/ {\vphantom {{n = \left( {z_{\alpha /2} + z_{\beta } } \right)^{2} } {2d^{2}_{\text{E}} }}} \right. \kern-0pt} {2d^{2}_{\text{E}} }}$$

where, *n* is the sample size; *z*, the value for the selected alpha level, for example 1.96 for (0.05), that is at 95%. Confidence level; 1−β, the statistical power; and *d*_E_, the effective size. It was decided to include 30 patients per group to provide for possible dropouts.

### Patients, outcome measures, and selection procedures

**Fig. 1:**
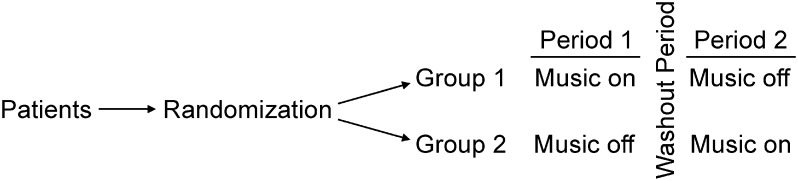
Two-sequence, two-period, and two-treatment crossover design

This was a randomized, two-sequence, two-period, and two-treatment crossover design [17, 18] where two treatments were consecutively administered in each patient. This study was carried out between May and December 2014. Approval for the research was obtained from the Institutional Review Board of the Catholic University of Korea, and all patients provided informed consent for participation in the study (PC145ISE0039). This study was conducted according to the guidelines of the Declaration of Helsinki. All procedures were performed by a single surgeon utilizing topical anesthesia. No systemic drugs (e.g., anxiolytics) were administered as adjuvant therapy (Fig. 1).

A total of 52 Korean patients with cataracts were randomly divided into two sequence groups; twenty-three patients in one group received the cataract surgery sequentially with and without KTM (in that order) and 29 patients in other group received the cataract surgery in the reverse sequence. The washout period between the first and second surgery was set at around 10 days according to the patients’ recovery conditions.

Eligibility criteria were as follows: willing to listen to music during surgery; and the absence of other ocular diseases, hearing disorders, or communication disorders.

The ages and cultural backgrounds of the patients were taken into account for selection of the particular song; the song was recorded with ‘*gayageum,*’ ‘*haegeum*’ (Korean traditional zithers), and an electronic keyboard. The song was chosen by a music expert and was played repeatedly during the procedure. The song had a simple melody without lyrics and with a limited dynamic range. It was played through earphones with an MP3 player during operation for patients in the KTM group. The patients were able to adjust the volume, and there was no interference with communication with the surgeon.

The primary endpoint was pain score, rated directly by the patients after surgery using the visual analog scale (VAS), in which a score of 0 indicated no pain, and a score of 10 indicated unbearable pain. As reference endpoints, systolic and diastolic blood pressures and pulse rates were assessed intra-operatively and postoperatively with the patients in the supine position (Patient Care Management System, Space Labs Medical, USA).

### Surgical procedure

For the surgical procedure, a continuous curvilinear capsulorhexis approximately 5.5 mm in diameter was made using forceps. Cataracts were removed by phacoemulsification through a 3.0-mm temporal corneal incision for the foldable intraocular lens. All intraocular lenses were implanted in the capsular bag. Postoperative topical therapy consisted of fluoroquinolone and steroid eye drops for 1 month.

### Statistical analysis

All data were analyzed using SAS for Windows (version 8.0.0). The significance level was 5%.

## Results

A total of 52 patients with bilateral cataracts were recruited to participate in the study. Patients ranged in age, with 13.5% of patients in their 50s, 26.9% of patients in their 60s, 50.0% of patients in their 70s, and 9.6% of patients in their 80s (Table 1). Women represented 63.5% of patients. Patients with high blood pressure comprised 63.5% of the total patient population, and 40.4% of patients had diabetes.

**Table 1:** Study sample statistics

Classification	Incidence (*n*)	Percent (%)
Sex		
Male	19	36.5
Female	33	63.5
Total	52	100
Age group (years)		
50s	7	13.5
60s	14	26.9
70s	26	50.0
80s	5	9.6
Total	52	100
Diabetes		
Yes	21	40.4
No	31	59.6
Total	52	100
High blood pressure		
Yes	33	63.5
No	19	36.5
Total	52	100

The mean pain scores on VAS were 3.15 ± 2.04 for patients in the KTM group and 4.11 ± 2.28 for patients in the non-KTM group. This difference was statistically significant (*p* = 0.013). Analysis of variance was used to determine whether there was a carryover effect in the crossover; this result failed to reach statistical significance (*p* = 0.0587; Table 2).

**Table 2:** Analysis of variance

Source	DF	Sum of squares	Mean squares	*F* value	*p* value
Between-subject					
Sequence (carryover)	1	20.2773	20.2773	3.74	0.0587
Residuals	50	270.8381	5.4168		
Within-subject					
Treatment	1	23.6928	23.6928	6.34	0.0151
Period	1	0.0005	0.0005	0.00	0.9906
Residual	50	186.9610	3.7392		
Total	103	502.1154			

Blood pressure and pulse comparisons for KTM and non-KTM cases are shown in Table 3. None of these reached statistical significance.

**Table 3:** Mean blood pressure and pulse measurements

Measurement	KTM	Non-KTM	*p* value
Blood pressure (mm Hg)			
Intra-operative systolic	149.37 ± 2.95	147.61 ± 2.28	0.512
Intra-operative diastolic	80.87 ± 1.54	78.02 ± 2.01	0.160
Postoperative systolic	140.42 ± 2.93	137.98 ± 2.97	0.312
Postoperative diastolic	76.48 ± 1.42	74.79 ± 1.39	0.162
Pulse (bpm)			
Intra-operative	74.13 ± 1.78	74.27 ± 1.78	0.731
Postoperative	68.65 ± 1.39	69.90 ± 1.57	0.178

## Discussion

The present study was designed to analyze the relationships between music stimuli and both pain and blood pressure during cataract surgery. Interestingly, we found that patients listening to KTM had significantly lower mean pain scores than patients who did not listen to music during the procedure.

In our study population, 63.5% of patients had hypertension, and 40.4% of patients had diabetes. These rates were significantly higher than corresponding disease rates for the general Korean population in equivalent age groups. According to Korean national survey results, 48.5% of men and 48.8% of women in their 60s and 59.0% of men and 64.3% of women over 70 have hypertension, while 28.5% of men and 22.1% of women in their 60s and 22.5% of men and 31.3% of women over 70 were diabetic [30].

Several studies have assessed pain experienced during cataract surgery comparing retrobulbar and topical anesthesia. Topical anesthesia has the advantage that sight recovery occurs on the day of surgery but is regarded as more painful during surgery than retrobulbar block [19, 20]. A comparative study of perceived pain for sequential surgery reported a pain score of 1 (range, 0–8) for the first eye surgery and 2 (range, 0–6) for the second eye surgery [21]. Another comparative study of perceived pain for sequential surgery reported the first eye and second eye surgery pain scores of 2.35 (SD = 2.63) and 2.89 (SD = 2.93), respectively, with no sedation [22]. Patients’ perceived pain during the second surgery was greater than that during the first surgery [21–23].

Several studies in this field have also been undertaken in Korea [19, 20, 23]. One such study reported an average pain score of 0.86 ± 0.55 with 3.24 ± 1.51 for the most painful surgery stage. In another study, pain score reported by women for the most painful stage was 3.43 ± 1.48 under topical anesthesia [23]. For a study comparing retrobulbar block and topical anesthesia, pain scores on a scale of 1 to 9 were divided into three sections and labeled as follows: 1–3: ‘light’; 4–6: ‘medium’; and 7–9: ‘severe’; the average pain reported fell into the ‘light’ category. All patients received diazepam (5 mg) orally before surgery [19]. A pain score of 4.75 ± 2.00 was reported in another study that did not involve administration of sedatives [20]. The VAS pain score difference in our study was statistically significant (KTM: 3.15, non-KTM: 4.11) irrespective of the surgery sequence; however, the scores were higher than those from other studies.

Emotional factors such as pain and anxiety affect the patients’ experiences of surgical procedures in ophthalmology more than that in other medical fields, and many studies have investigated measures for managing such emotional factors [5, 7, 16]. Because loss of vision is one of the most feared disabilities [1], people are highly concerned about any procedures involving their eyes. Cataract surgeries are performed under local or topical anesthesia, and patients’ cooperation is an important factor for a successful procedure. The ability of patients to interact with the surgeon and patients’ perceived pain and anxiety are all contributing factors [22, 24].

Several types of sedatives, including midazolam, dexmedetomidine, propofol, and benzodiazepines, may cause oversedation, interfering with the patients’ ability to cooperate during surgery and affecting recovery time postoperation [25, 26]. Furthermore, elderly patients experience a greater risk of adverse effects of drugs than younger individuals due to differences in drug metabolism [4, 16]. As such, adjuvant methods, in addition to sedatives, may also be useful for reducing pain and increasing patient satisfaction of surgical experience.

A previous study reported the effects of music on reducing anxiety and pain in patients undergoing intravitreal injections [25] and found that music had positive effects on reducing anxiety levels in patients prior to administration of the injection. Notably, intravitreal injection requires only a few minutes, while cataract surgery requires between 15 and 35 min. This may explain the differences in perceived pain between the two studies. Furthermore, because patients may undergo intravitreal procedures on multiple occasions, patients may have preconceived expectations of pain for subsequent procedures, which may affect the perceived pain [6, 21].

Studies in other medical fields have examined the effects of music on various indicators of pain and anxiety. For example, patients who had undergone surgery with general anesthesia and required longer recovery times after the procedure or were hospitalized showed positive responses to engagement of music, e.g., less pain and reduced fatigue after discharge following the procedure [12, 27].

Moreover, a number of previous studies have shown that ethnicity is an independent variable affecting their results [6, 28]. In this study, KTM as an adjuvant method was shown to be effective in reducing pain in patients undergoing cataract surgery. East Asians, particularly Koreans, react more acutely to indirect, implied, or transformational signs than Americans, who show a greater inclination to react to informational signs [29]. Consequently, pain felt by ethnic groups categorized as ‘high-context’ may be more effectively managed through the use of music. In particular, the Korean elderly population relates more naturally to KTM, which is intertwined with Korean cultural context. This allows enhanced effective management of pain associated with cataract surgery. Consistent with this, we hypothesized that the use of KTM would have a positive enhancing effect on the surgical experience of Korean patients with cataracts. The result showed that there was a positive correlation between KTM and pain management. The majority of patients who had cataract surgery with KTM indicated that they would prefer to have music played in future medical procedures.

A unique aspect of ophthalmic surgery is its symmetry. Differences between subjects in pain perception may introduce a bias in the results obtained. The crossover design utilized in this study is useful for eliminating interperson bias as the study involved two sequential procedures performed on one patient, and no carryover effect was observed.

## Limitations

Unlike measurements such as weight and blood pressure which can be objectively measured, perceived pain is a subjective indicator. Therefore, interviews with subjects after the operation may have been required to discover whether the comparatively high pain score recorded in this study was a result of the surgery method used or due to perceived differences of the pain score scale used.

## Conclusion

To the best of our knowledge, this is the first study to analyze the effects of music therapy during cataract surgery while accounting for cultural differences (i.e., using KTM for the Korean population), giving our analysis a patient-centered approach. Other studies have shown relationships between music and pain and/or anxiety; however, this study was designed to evaluate the effects of KTM on pain utilizing a crossover design. This study showed that KTM had a statistically significant effect on reducing pain during cataract surgery regardless of the sequence and that KTM was more effective for patients with high blood pressure. This finding may be translatable to other surgeries performed on Korean patients under local anesthesia; however, further studies are required.
